# Molecular Docking Studies on the Anti-viral Effects of Compounds From Kabasura Kudineer on SARS-CoV-2 3CL^pro^

**DOI:** 10.3389/fmolb.2020.613401

**Published:** 2020-12-23

**Authors:** Savariar Vincent, Selvaraj Arokiyaraj, Muthupandian Saravanan, Manoj Dhanraj

**Affiliations:** ^1^Centre for Environmental Research and Development (CERD), Loyola College, Loyola Institute of Frontier Energy, Chennai, India; ^2^Department of Food Science and Biotechnology, Sejong University, Seoul, South Korea; ^3^Division of Biomedical Science, Department of Medical Microbiology and Immunology, School of Medicine, College of Health Sciences, Mekelle University, Mekelle, Ethiopia

**Keywords:** COVID-19, molecular docking, Kabasura kudineer, iGemdock, anti-viral effects

## Abstract

The COVID-19 has now been declared a global pandemic by the World Health Organization. No approved drug is currently available; therefore, an urgent need has been developed for any antiviral therapy for COVID-19. Main protease 3CL^pro^ of this novel Coronavirus (SARS-CoV-2) play a critical role in the disease propagation, and hence represent a crucial target for the drug discovery. Herein, we have applied a bioinformatics approach for drug repurposing to identify the possible potent inhibitors of SARS-CoV-2 main proteases 3CL^pro^ (6LU7). In search of the anti-COVID-19 compound, we selected 145 phyto-compounds from Kabasura kudineer (KK), a poly-herbal formulation recommended by AYUSH for COVID-19 which are effective against fever, cough, sore throat, shortness of breath (similar to SARS-CoV2-like symptoms). The present study aims to identify molecules from natural products which may inhibit COVID-19 by acting on the main protease (3CL^pro^). Obtained results by molecular docking showed that Acetoside (−153.06), Luteolin 7 -rutinoside (−134.6) rutin (−133.06), Chebulagic acid (−124.3), Syrigaresinol (−120.03), Acanthoside (−122.21), Violanthin (−114.9), Andrographidine C (−101.8), myricetin (−99.96), Gingerenone -A (−93.9), Tinosporinone (−83.42), Geraniol (−62.87), Nootkatone (−62.4), Asarianin (−79.94), and Gamma sitosterol (−81.94) are main compounds from KK plants which may inhibit COVID-19 giving the better energy score compared to synthetic drugs. Based on the binding energy score, we suggest that these compounds can be tested against Coronavirus and used to develop effective antiviral drugs.

## Introduction

The World Health Organization has declared novel Coronavirus disease 2019 (COVID-19) to be a pandemic that went on to affect more than 219 countries with 44,002,003 confirmed cases and killed more than 1,167,988 people (WHO as of Oct 29, 2020) (Velavan and Meyer, 2020). The updated information is available on the WHO website (https://www.who.int/emergencies/diseases/novel-coronavirus-2019). It has also ignited fears of an impending economic crisis and recession in the infected countries (Buck et al., 2020). In India, the first case was reported on January 30, 2020; as of Oct 29, 2020; 8,040,203 cases have been confirmed by COVID-19 infection along with 7,032,000 recoveries, 120,527 deaths with the fatality rate of 1.2% (Ministry of Health and Family Welfare, India). On March 25, 2020, the Government of India announced a nationwide lockdown to cut the chain of community transmission. COVID-19 is caused by Severe Acute Respiratory Syndrome Coronavirus 2 (SARS-CoV2) which results in respiratory illness among infected people. There are 7 Human Corona Virus (HCoV) strains identified so far and categorized into α-CoV (229E and NL63) and β-CoV (OC43, HKU1, SARS, MERS, and COVID-19 HCoVs). Among these, MERS HCoV and SARS were reported to be more virulent and have the highest mortality (Elfiky, 2020).

The HCoV is a positive sense virus with a single-stranded 30,000 bp RNA (+ssRNA). The virus consists of two clusters of proteins, namely (a) the non-structural RNA-dependent RNA polymerase (RRP) that is significant in the replication of the virus, and 3C-Like Protease (3CL^pro^) enzyme that cleaves the two polyproteins (PP1A and PP1AB) translated from viral RNA in the host cell, and (b) Spike proteins that help in fusion and entry of the virus into the host, Nucleocapsid, Matrix and Envelope proteins (Elfiky et al., 2017).

The major symptoms include fever, cough, and breathing difficulties. So far, no vaccine is available, and no drugs have been found to cure the life-threatening coronavirus infection. But the research is continuing to identify the potent drug or vaccine. Only symptomatic relief is provided to the patients. Currently, antiviral drugs, MERS-Cov antibodies, SARS-CoV, and combination therapy of hydroxychloroquine and azithromycin are recommended (Gautret et al., 2020; Huang et al., 2020). These compounds prevent viral entry by inhibition of Angiotensin-Converting Enzyme 2 (ACE2) cellular receptor, acidification of cell membrane, and by immunostimulant activity. However, some reports show that the drug hydroxychloroquine is not effective for those infected with the coronavirus and shows adverse effects in patients with acute renal impairment (Pelle and Callen, 2002; Tailor et al., 2012).

Therefore, there is an urgent need to develop an alternative method to prevent novel SARS-CoV2 infection. Siddha medicine, an Indian medical system, uses specific polyherbal formulations for the treatment of infectious diseases (Zysk, 2009; Rajantheran et al., 2014).

Traditional methods of drug discovery could take years, whereas *in silico*-docking analysis enables large-scale screening fast, reliable, and cheaper than conventional drug development (Green and Segall, 2014). *In silico* analysis, we assess the binding ability of a ligand to protein at an active site as well as to compare the binding modes of different ligands to the active site-pocket (Leach, 2001). In this study, we used 3CL^pro^ as a possible targeting site to treat HCoV. Previously the main protease (Mpro)/chymotrypsin-like protease (3CL^pro^) has been isolated through crystallization by Liu and Wang (2020) (PDB ID: 6LU7). The mechanism of 3CL^pro^ is deciphered computationally as the stearic interaction with glycine in the polyproteins and forms a strong hydrogen bonding to stabilize the complex. A conserved GSCGS motif has been observed to form three consecutive turns which were consequently temporarily stabilized by PNCC. This stabilizing PNCC is located on the surface opposite to the active site and hence can be the potential drug targeting site for 3CL^pro^ inhibitors (Wang et al., 2020).

In search of the anti-COVID-19 compound, we selected 145 phyto-compounds from Kabasura kudineer, a poly-herbal formulation prescribed in AYUSH for COVID-19 which are effective against flu, cough, sore throat, shortness of breath (similar to SARS-CoV2 like symptoms). [AYUSH Ministry of Health Corona Advisory—D.O. No. S. 16030/18/2019—NAM; 06th March 2020]. Therefore, the present study involves the analysis of 145 phytocompounds from Kabasura kudineer against the structure of SARS-CoV-2 3CL^pro^ through structure-based *in silico* molecular docking and to identify potent anti-COVID-19 natural compounds.

## Materials and Methods

*In silico* docking of the protein SARS-CoV-2 virus 3CL^pro^ (PDB ID: 6LU7), iGEMDOCK module software was used.

### Selection of Protein

**Figure 1:**
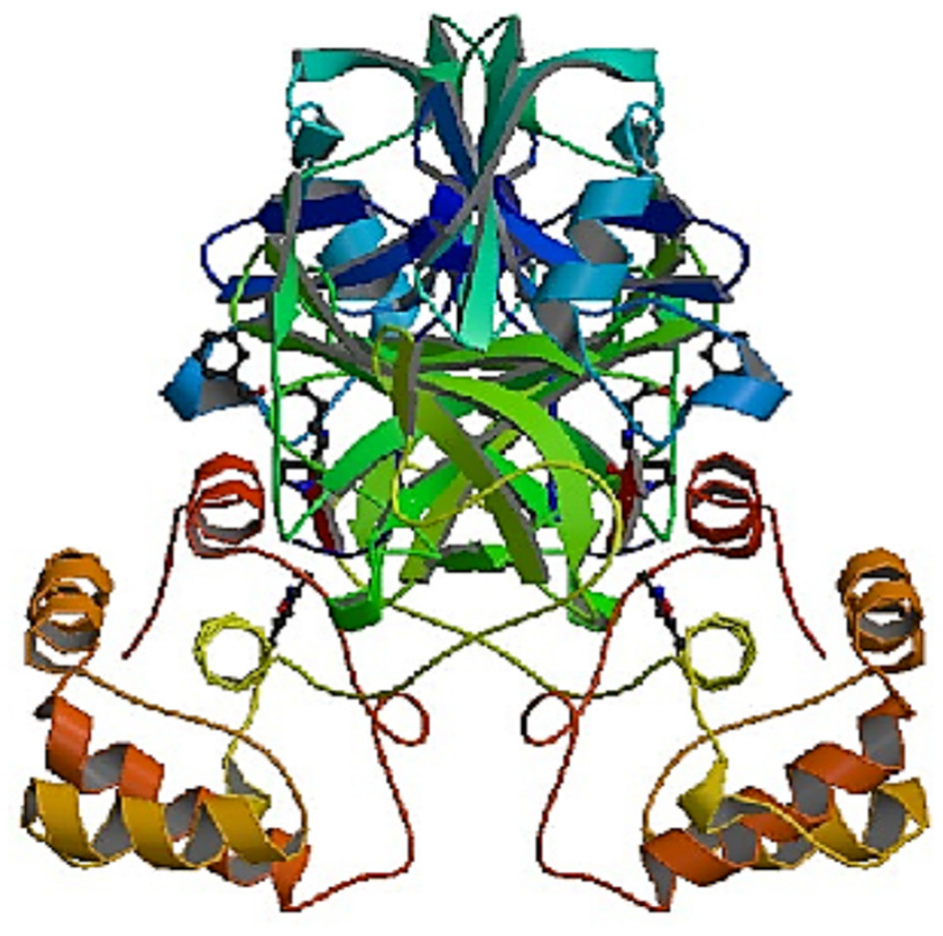
The crystal structure of COVID-19 main protease in complex with an inhibitor N3 PDB ID: 6LU7.

The 3CL^pro^/Mpro (PDB ID: 6LU7) protein structure COVID-19 (Figure 1) containing the two chains (A&B) was acquired from the protein data bank (www.rcsb.org). The PDB format extraction was used to study the crystal structure of the protein.

### Ligand Preparation

We selected 145 phyto-compounds from Kabasura kudineer complex (Supplementary Table 1). A total of 145 two-dimensional ligands were generated with ACD chem sketch. The ligands extracted in the mol format were converted to pdb format using OPEN BABEL software (www.vcclab.org/lab/babel/start.html). The docking tool iGEMDOCK v2.0 was used for the rapid virtual screening of the extracted compounds (Yang and Chen, 2004). Based on the highest energy value, best compounds from each plant of Kabasura kudineer complex were exposed to accurate molecular docking (Supplementary Table 2).

### Active Site Prediction

The most significant step in molecular docking is to locate the ligand-binding sites on a protein. The protein-ligand binding sites are located using the novel energy-based method Q-Site Finder developed by Jackson, where the interaction energies of a methyl probe with a protein are analyzed (Laurie and Jackson, 2005). Using the software, the plausible active binding sites of toxins were obtained. The binding sites which are more flexible were preferred for this analysis.

### Protein-Ligand Docking

After the compounds are screened, a virtual screening environment is created through an integrated tool iGEMDOCK. The tool affords the interactive interfaces to prepare both the binding site of the target protein and the screening compound library. Post-screening, each compound in the library is docked into the binding site and thereby generating the protein-compound interaction profiles of electrostatic, hydrogen-bonding, and van der Waals interactions. The tool then corroborates the pharmacological interactions and clusters the screening compounds for the post-screening analysis based on the interaction data and the compound structures, ranks and visualizes the screening compounds by combining the pharmacological interactions and energy-based scoring function. With the population size 800 set with 80 generation and 10 solutions, the ligands were subjected to very slow accurate docking and the docking pose and their corresponding energy values were analyzed.

## Results and Discussion

The present study focused on the main proteases 3CL^pro^ PDB ID (6LU7), as potential target proteins for SARS - COVID-19. Inhibition potential and effectiveness of compounds from Kabasura kudineer (KK) against novel coronavirus 3CL^pro^ on molecular docking studies using Igemdock were investigated. The proteolytic maturation enzyme Mpro in CoV has been identified as the potential target protein to prevent the spread of infection through the inhibition of viral polyprotein cleavage (Liu and Wang, 2020). The discovery of the Mpro protease structure has facilitated the identification of potential drug candidates for treatment. The protein sequences of the two Coronavirus strains SARS-CoV Mpro and the 2019-nCoV Mpro are 96% identical, and the active sites in both proteins remain free from mutations. In many viruses, proteases play essential roles in viral replication; therefore, proteases are often used as potential targets during the development of antiviral therapeutics (Chang et al., 2019). However, the disruption of protease activity in host cells can lead to various diseases. Hence, the host proteases can be generally used as potential therapeutic targets. Several compounds from medicinal plants have been reported to show antiviral properties (Thayil and Thyagarajan, 2016; Zakaryan et al., 2017; Jo et al., 2020). This study was conceived with a strategy of exploring the natural compounds which may impede the SARS-CoV-2 infection by blocking the viral entry into the host cell or inhibiting the viral polyprotein processing in the cell. In this context, KK comprises 15 individual herbs namely *Andrographis paniculata, Syzygium aromaticum, Zingiber officinale, Tragia involuerta, Hygrophila auriculata, Terminaila chebula, Adhatoda vasica, Coleus ambonicus, Saussurea lappa, Clerodendrum serratum, Cypreus rotundus, Tinospora cordifolia, Sida acuta, Piper longum*, and *Anacyclus pyrethrum*. Each plant consists of several compounds and exhibits various biological activities (Table 1).

**Table 1:** List of plants included in Kabasura kudineer, a poly herbal formulation as recommend by AYUSH.

**S. No**	**Medicinal plants**	**Pharmacological properties**	**References**
1	*Andrographis paniculata*	Anti-viral, anti-cancer, anti-inflammatory, angiogenic, anti-venom, anti-diabetic, and anti-malarial properties	Bharati et al., 2011; Hossain et al., 2014
2	*Syzygium aromaticum*	Anti-bacterial, anti-fungal, insecticidal, analgesic, anti-spasmodic, anti-carminative, and anti-oxidant properties	Jimoh et al., 2017; Kaur and Kaushal, 2019; Batiha et al., 2020
3	*Zinigiber officianle*	Anti-emetic, anti-oxidant, anti-diabetic, anti-pyretic, analgesic, anti-arthritic, and anti-inflammatory activities	Amir et al., 2011; Rahmani, 2014; Marrelli et al., 2015
4	*Tragia involucrata*	Anti-microbial, analgesic, anti-epileptic, anti-diabetic, anthelmintic, anti-inflammatory, anti-fertility, anti-diuretic, anti-pyretic, anti-oxidant, and cytotoxic activities	Dhara et al., 2000; Rao et al., 2007; Mothana et al., 2010; Gobalakrishnan et al., 2013
5	*Hygrophila auriculata*	Anti-cancer, hypoglycemic, aphrodisiac, anti-microbial, anti-oxidant, lipid peroxidation, hepatoprotective, and hematopoietic activity	Hussain et al., 2010; Salve and Bhuktar, 2017
6	*Terminalia chebula*	Anti-bacterial, anti-microbial, anti-fungal, anti-viral, anti-oxidant, anti-ulcer, and anti-helmintic	Ashwini et al., 2011; Rathinamoorthy and Thilagavathi, 2014; Upadhyay et al., 2014
7	*Adhatoda vasica*	Anti-asthmatic and bronchodilator activity, wound healing activity, anti-ulcer activity, cholagogue activity, anti-allergy activity, anti-tubercular activity, abortifacient and uterotonic activity, insecticidal activity, and anti-bacterial activity	Singh et al., 2011; Gangwar and Ghosh, 2014; Kumar et al., 2014
8	*Coelus amboinicus*	Urolithiasis, fungitoxic, anti-bacterial, anti-malarial, and anti-inflammatory	Pillai et al., 2011; Rout et al., 2012; Arumugam et al., 2016
9	*Saussurea lappa*	Anti-arthritic, anti-convulsant, anti-cancer, anti-inflammatory, anti-larvicidal, anti-ulcer, anti-viral, and hepatoprotective activities	Liu et al., 2012; Madhuri et al., 2012; Singh et al., 2017
10	*Clerodendrum serratum*	Anti-oxidant, anti-bacterial, anti- carcinogenic, hepatoprotective, wound healing, and anti-allergic properties	Patel et al., 2014; Noreen et al., 2018; Mahajan et al., 2019
11	*Cyperus rotundus*	Anti-androgenic, anti-mutagenic, anti-obesity, anti-oxidant, anti-uropathogenic, anti-diarrheal, anti-genotoxic, anti-cancerous, anti-convulsant, anti-diabetic, anti-bacterial, anti-lipidemic, antimalarial, anti-inflammatory, hepatoprotective, cardioprotective, and neuroprotective	Peerzada et al., 2015; Al-Snafi, 2016
12	*Tinospora cordifolia*	Anti-cancer, anti-diabetes, anti-viral, anti-inflammatory, anti-psychiatric, and immunomodulatory action	Rout, 2006; Patel et al., 2009; Upadhyay et al., 2010; Gupta and Sharma, 2011; Tiwari et al., 2018
13	*Sida acuta*	Anti-plasmodial, anti-ulcer, hypoglycemic, anti-bacterial, anti-fungal, anti-oxidant, anti-inflammatory, analgesic, anti-pyretic, hepatoprotective, and cytotoxic activities	Karou et al., 2017; Jindal et al., 2012; Tcheghebe et al., 2017
14	*Piper longum*	Anti-cancer, anti-oxidant, anti-inflammatory, anti-microbial, anti-hyperlipidemic, anti-obesity, and analgesic activities	Zaveri et al., 2010; Kumar et al., 2011
15	*Anacyclus pyrethrum*	Anti-rheumatic, analgesic, anti-bacterial, anti-diabetic, anti-oxidant, anti-inflammatory, and anti-nociceptive activities	Selles et al., 2013; Usmani et al., 2016

*Ref: AYUSH Ministry of Health Corona Advisory—D.O. No. S. 16030/18/2019—NAM; dated: 06th March, 2020*.

From the documents of Dr. Lipinski, the molecules are categorized as the therapeutic compounds when they have sufficiently acceptable ADME properties (absorption, distribution, metabolism and excretion) and toxicity profiles to qualify through the Phase I clinical trial on humans. However, his postulate “rule of 5” classifies the molecules only based on the orally active drug phenomena that include molecular weight ≤ 500, clogP ≤ 5, H- bond donor ≤ 5, and H- bond acceptor ≤ 10. They do not evaluate the parameters of direct metabolism, frequency of the molecule or if it contains reactive functional groups.

In this present study, SARS-CoV-2 virus 3CL^pro^ was docked with 145 compounds selected from KK comprising plants. Ritonavir, Lopinavir, Oseltamivir, HCQ, Ivermectin, and Azithromycin are used as standard (Caly et al., 2020; Cao et al., 2020; Muralidharan et al., 2020). These ligands were screened based on their propensity to dock with the receptor molecule and to inhibit the protein activity. The extent of docking was ranked based on the iGEMDOCK scoring function to zero down the most accurate ligand. Eventually, during the virtual screening process, each compound from each plant that bound at different binding pockets of the 3CL^pro^ has been selected. Based on the highest energy value, best compounds from each plant of Kabasura kudineer complex were exposed to accurate molecular docking. Based on screening process, the highest-ranked compounds (Acetoside, Luteolin 7 –rutinoside, Rutin, Chebulagic acid, Acanthoside, Syrigaresinol, Violanthin, Andrographidine C, Myricetin, Gingerenone -A, Tinosporinone, Geraniol, Nootkatone, Asarianin, and Gamma sitosterol) were selected for accurate docking against SARS-CoV-2 virus 3CL^pro^ and their corresponding energy values are listed in Table 2 (Structure of target compounds are shown in Supplementary Figure 1). The energy values are inversely proportional to the acceptability of the molecule as a drug. Molecules that scored best by iGEMDOCK scoring functions were identified as potential leads for COVID-19 drug discovery process.

**Table 2:** Accurate Molecular Docking studies on the target Phytocompounds from Kabasura kudineer (KK) with 3CL^pro^ (6LU7) of the SARS-CoV2 Coronavirus using iGEMDOCK software.

**S. no**	**Name of the plant**	**Compound name**	**Molecular formula and weight**	**Energy**	**VDW**	**H bond**	**Binding domain**	**Aminoacid**
1	*Clerodendrum serratum*	Acetoside (S. Figure 1a)	C_29_H_36_O_15_ 624.6 g/mol	−153.06	−93.6	−59.46	H-S^*^ H-M V-M V-S	**ARG-131** THR-199 ASN-238 LEU-287 ASP-289 THR-199 TYR-239 LEU-286 LEU-287 ASP-289
2	*Hygrophila auricualata*	Luteolin 7 -rutinoside (S. Figure 1b)	C_27_H_30_O_15_ 594.5 g/mol	−134.6	−98.39	−36.23	H-S H-M V-M^*^ V-S	ARG-131 THR-199 ASN-238 **LEU-287** ASP-289 THR-199 TYR-239 LEU-286 LEU-287 ASP-289
3	*Tragia involerta*	Rutin (S. Figure 1c)	C_27_H_30_O_16_ 610.5 g/mol	−133.06	−85.08	−47.99	H-S H-M V-M V-S	ARG-131 THR-199 LEU-287 THR-199 TYR-239 LEU-286 LEU-287 ASP-289
4	*Terminalia chebula*	Chebulagic acid (S. Figure 1d)	C_41_H_30_O_27_ 954.7 g/mol	−124.3	−103.2	−21.28	H-S H-M V-M V-S^*^	THR-199 LEU-287 ASP-289 THR-199 **TYR-239** LEU-286 LEU-287 ASP-289
5	*Sida acuta*	Acanthoside (S. Figure 1f)	C_34_H_46_O_18_ 742.7 g/mol	−122.21	79.44	−42.5	H-S^*^ H-M V-M V-S	**ARG-131** THR-199 LEU-287 TYR-239 LEU-286 LEU-287 ASP-289
6	*Sausurea lappa*	Syrigaresinol (S. Figure 1e)	C_28_H_36_O_13_ 580.6 g/mol	−120.03	−79.99	−40.04	H-S^*^ V-S	ASN-151 **GLU-240** THR-292 GLY-110
7	*Adhatoda vasica*	Violanthin (S. Figure 1g)	C_27_H_30_O_14_ 578.5 g/mol	−114.9	−76.13	−38.81	H-M H-S^*^ V-M	PHE-219 ASN-221 **LEU-271** ARG-279 LEU-220
8	*Andrographis paniculata*	Andrographidine C (S. Figure 1h)	C_23_H_24_O_10_ 460.4 g/mol	−101.8	−72.41	−29.36	H-S H-M V-M^*^ V-S	ARG-131 THR-199 ASN-238 ASP-289 THR-199 TYR-239 **LEU-286** LEU-287 ASP-289
9	*Syzygium aromaticum*	Myricetin (S. Figure 1i)	C_15_H_10_O_8_ 318.23 g/mol	−99.96	−63.64	−36.32	H-S^*^ V-S	**ARG-188** GLU-55
10	*Zingiber Officianle*	Gingerenone -A (S. Figure 1j)	C_21_H_24_O_5_ 356.4 g/mol	−93.9	−69.96	−23.94	H-S H-M V-M^*^ V-S	ARG-131 THR-199 **LEU-287** ASP-289 THR-199 TYR-239 LEU-286 LEU-287 ASP-289
11	*Tinospora cordifolia*	Tinosporinone (S. Figure 1k)	C_19_H_18_O_6_ 342.3 g/mol	−83.42	−76.55	−6.86	H-S V-M V-S^*^	THR-199 ASP-289 **THR-199** TYR-239 LEU-287
12	*Ceolus ambonicus*	Geraniol (S. Figure 1l)	C_10_H_18_O 154.25 g/mol	−62.87	−57.1	−5.6	H-M V-M^*^	**THYR-26** LEU-141 ASN-142 LEU-4
13	*Cypreus rotundus*	Nootkatone (S. Figure 1m)	C_15_H_22_O 218.33 g/mol	−62.4	−52.51	−9.85	H-S^*^ V-S	**THY 111** THY- 292 ASP- 295 PHE-294
14	*Piper longum*	Asarianin (S. Figure 1n)	C_20_H_18_O_6_ 354.4 g/mol	−79.94	−71.92	−8.02	H-S^*^ V-M	TYR-239 LEU-286 LEU-287
15	*Anacyclus pyrethrum*	Gamma sitosterol (S. Figure 1o)	C_29_H_50_O 414.7 g/mol	−81.94	−79.44	−2.5	H-S **V-M**	ALA−70 ASP-289 THR-199 TYR-239

* *The possible binding modes of selected phytochemicals at the target protein active sites*.

*H-S signifies hydrogen bond with sidechain*.

*H-M signifies hydrogen bond with the main chain*.

*V-M signifies Vander waals interaction with main chain*.

*V-S signifies Vander waals interaction with side chain*.

*S. Figure – Supplementary Figure 1 (a–o)*.

*Bold values indicates the best interaction of amino acids along with hydrogen bond, side chain, main chain, and vander walls*.

**Figure 2:**
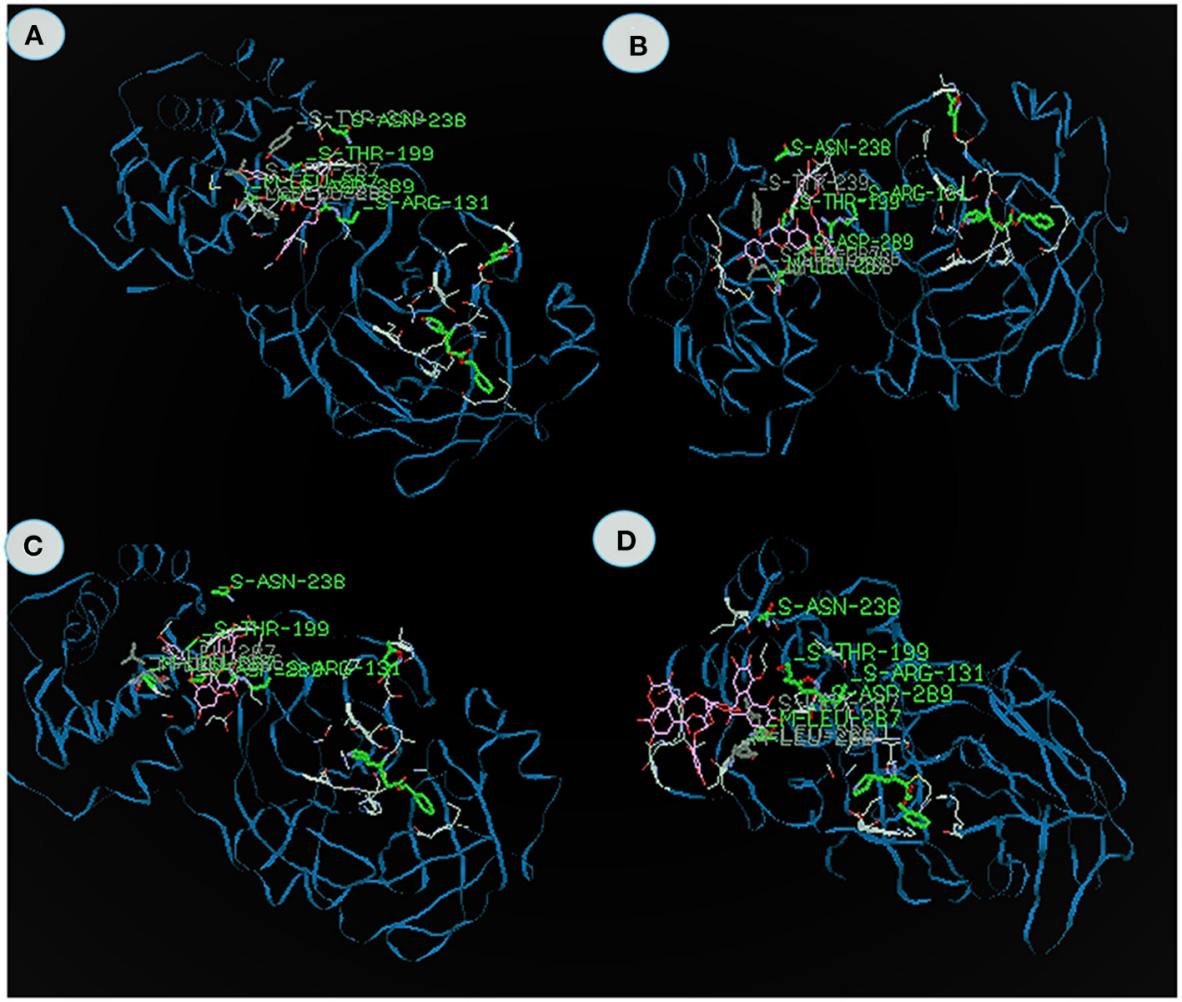
The binding affinities of target compounds **(A)** Acetoside, **(B)** Luteolin 7 -rutinoside **(C)** rutin **(D)** Chebulagic acid present in the Kabasura kudineer (KK) with 3CL^pro^ (6LU7) of the SARS-CoV2 Coronavirus.

Table 2 shows the energy value, binding domain and amino acids found in the active site pockets of 6LU7 against compounds from KK. The Acetoside from *Clerodendrum serratum* docked with the 3CL^pro^ and the total fitness value was found to be −153.06 kcal/mol, which comprises of −93.6 van der Waal interactions and −59.46 kcal/mol hydrogen bonding interactions. The inhibitor closely fits the active site cavity making various close contacts with the residues including hydrogen bonding with the main chain of arginine at position 131 with binding energy value −9.3 kcal/mol (Figure 2A). Acteoside, a phenylethanoid glycoside, is an active compound in several plants and traditional herbal medicines (Kubica et al., 2017). The study reports that the acetoside having the highest binding energy is very effective to inhibit the 3CL^pro.^ Similarly, Song et al. (2016) reported that the acetoside inhibits viral infections in a dose-dependent manner. The interactions and fitness score of the compound suggest that these leads can be formulated as an anti-COVID drug.

Luteolin 7- rutinoside from *Hygrophila auricualata* docked with 3CL^pro^ exhibits the energy value −134.6 kcal/mol, which comprises of −98.9 van der Waal interaction and −36.23 kcal/mol hydrogen bonding interactions (Figure 2B). Here, the main chain Leucine at position 287 with binding energy is −11.8 kcal/mol. It was predicted that the hydroxyl (-OH), ketone (=O), and ether (-O-) groups in luteolin compounds majorly impact the amino acid residue interactions at the active site of COVID-19 Mpro (Aziz et al., 2018). Luteolin derivatives have been predicted to interact with the NS2/NS3 protease used *in silico* analysis showed that luteolin reduces DENV infection through the inhibition of human furin, which is an enzyme involved in the maturation of the virions (Ramana et al., 2015; Dwivedi et al., 2016). It is apparent from the study, that Luteolin 7- the 3CL protease.

Rutin from *Tragia involerta* docked with 3CL^pro^ exhibits the energy value is −133.06 kcal/mol, which comprises of −85.08 van der Waal interaction and −47.99 kcal/mol hydrogen bonding interactions (Figure 2C). Here, the main chain is arginine with H-S domain at the position 131 with binding energy −11.8 kcal/mol. Jasso-Miranda et al. (2019) and Zandi et al. (2011) studied the antiviral activity of Rutin against Dengue virus type-2.

Chebulagic acid from *Terminalia chebula* docked with 3CL^pro^ exhibits the energy value of −124.3 kcal/mol, which comprises of −103.02 van der Waal interaction and −21.28 kcal/mol hydrogen bonding interactions (Figure 2D). The inhibitor closely fits the active site cavity making various close contacts with the residues including hydrogen bonding with the main chain of Thyronine at position 239 with binding energy value −17.9 kcal/mol. Li et al. (2020) observed chebulagic acid as Novel Influenza Viral Neuraminidase Inhibitor. Similarly, Lin et al. (2013) also observed the chebulagic acid blocked herpes simplex virus type 1 (HSV-1).

**Figure 3:**
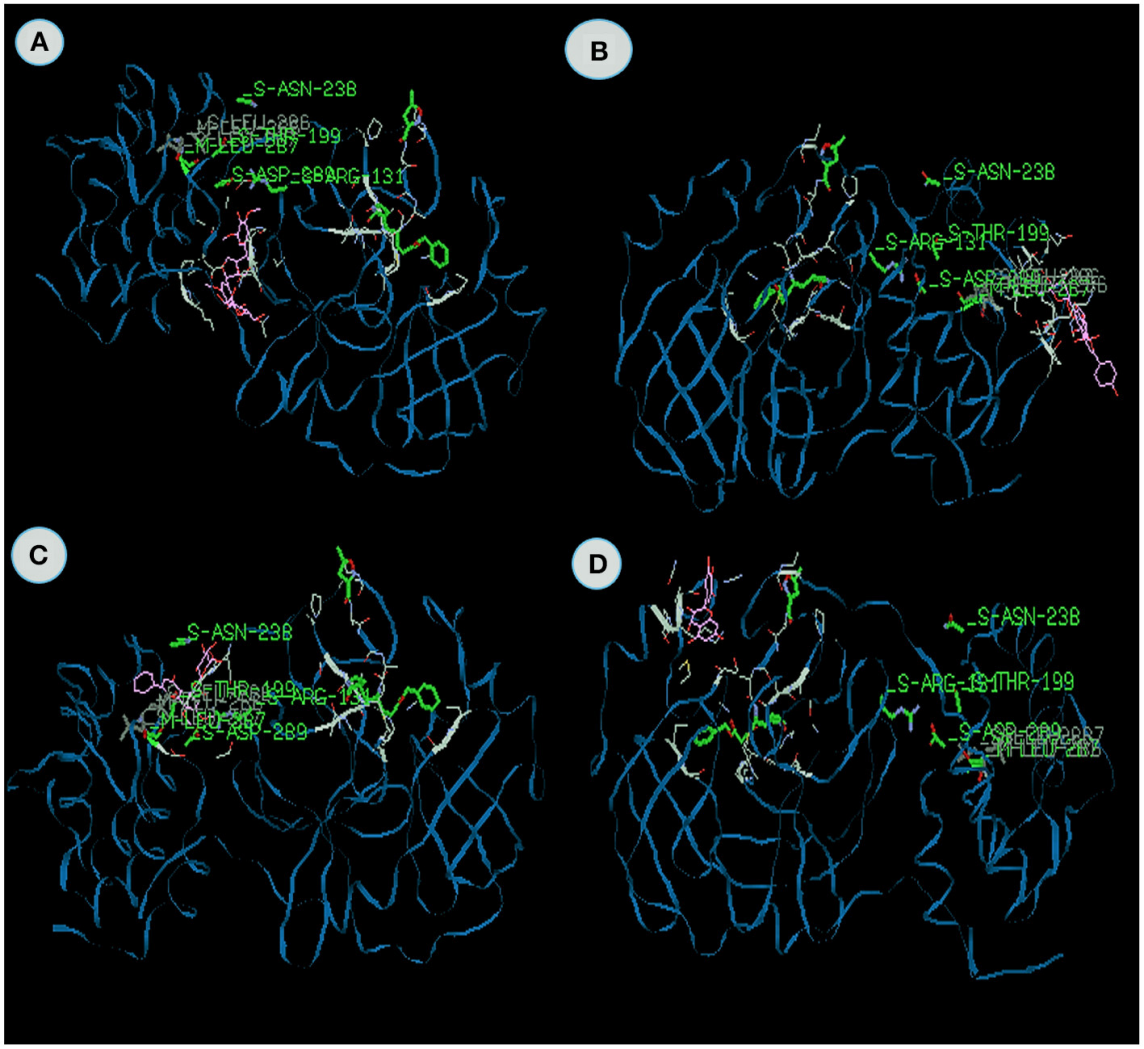
The binding affinities of target compounds **(A)** Syrigaresinol **(B)** Acanthoside **(C)** Violanthin **(D)** Andrographidine C present in the Kabasura kudineer (KK) with 3CL^pro^ (6LU7) of the SARS-CoV2 Coronavirus.

Acanthoside isolated from *Sida acuta* possesses the energy value −122.21 kcal/mol, which contains the −79.44 van der Waal interaction and −42.5 kcal/mol hydrogen bonding interactions (Figure 3B). Arginine 131 is the major active site to bind with receptors and the domain of H-S. *Sida* genus possesses various biological activities, especially anti-viral properties. No anti-viral reports have been reported from Acanthoside from *Sida acuta*.

Docking value of Syrigaresinol (Figure 3A) from *Sausurea lappa* exhibits −120.03 kcal/mol, Violanthin from *Adhatoda vasica* exhibits −114.9 kcal/mol on 3CL^pro^ which comprises of −79.99 and −76.13 van der Waal interactions and −40.04 and −38.81 kcal/mol hydrogen bonding interactions (Figure 3C). The residues include hydrogen bonding with the main chain of Glucine at position 240 with binding energy value −7.6 kcal/mol in Syrigaresinol and Leucine at position 271 with binding energy value −8.2 kcal/mol in Violanthin respectively. Likewise, Ouyang et al. (2007) also studied the Syringaresinol lignan inhibiting the multiplication of the tobacco mosaic virus. No study has been reported on violanthin, but this plant exhibits various pharmacological activities. It is evident from the study both the compounds effectively inhibited the 3CL protease.

Andrographidine C from *Andrographis paniculata* docked with 3CL^pro^ exhibits the energy value of −101.8 kcal/mol, which comprises of −72.41 van der Waal interactions and 29.36 kcal/mol hydrogen bonding interactions (Figure 3D). Here, the main chain and the amino acid region is leucine with V-M domain at the position 286 with binding energy −8.3 kcal/mol. Wiart et al. (2005) reported Antiviral properties of ent-labdene diterpenes of *Andrographis paniculata* Nees, inhibitors of herpes simplex virus type 1. Similarly, Sharma et al. (2019) also studied that the Green synthesis of silver nanoparticles from *Andrographis paniculata* exhibits the antiviral potential against chikungunya virus.

**Figure 4:**
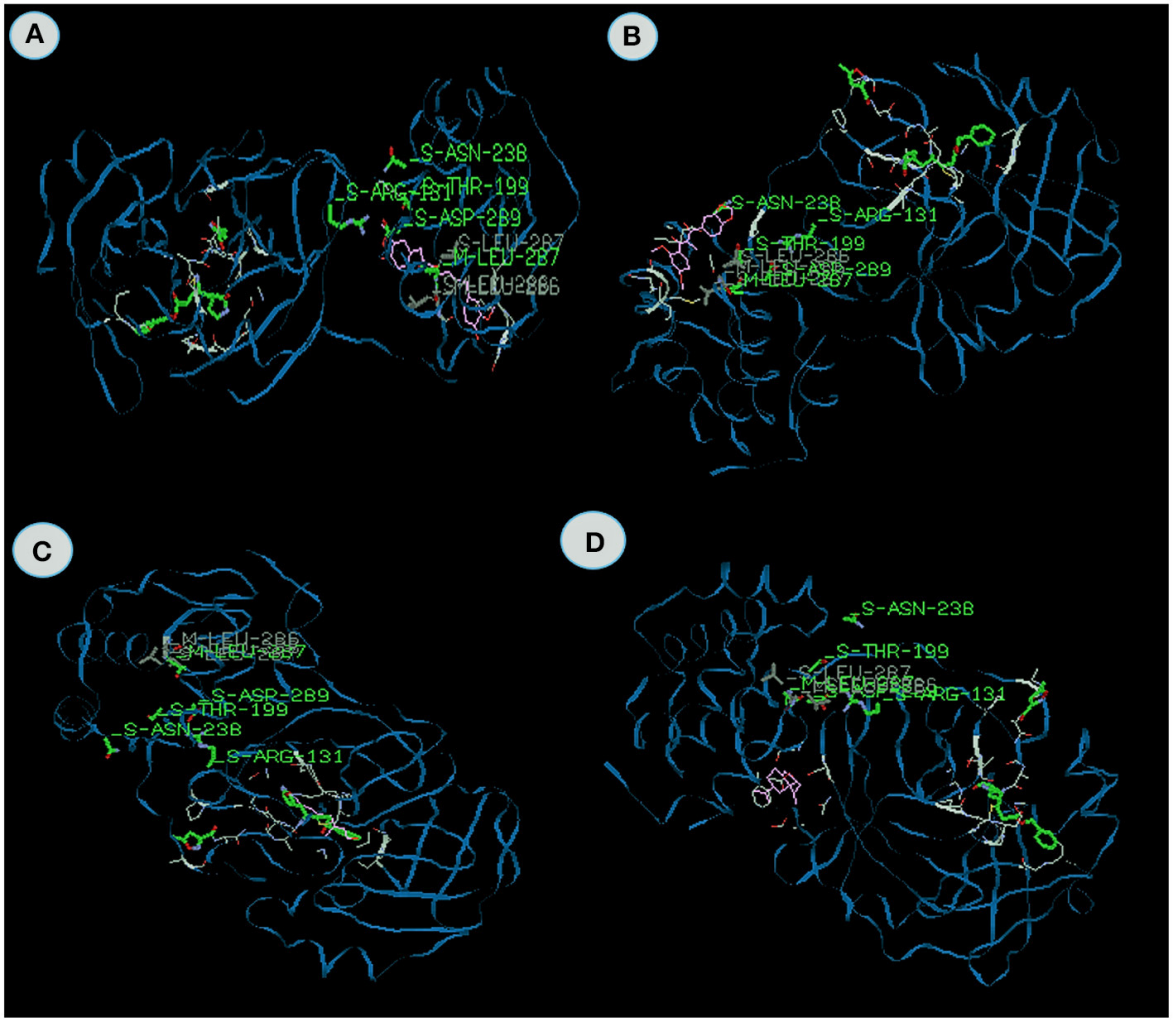
The binding affinities of target compounds **(A)** myricetin **(B)** Gingerenone -A, **(C)** Tinosporinone, **(D)** Geraniol present in the Kabasura kudineer (KK) with 3CL^pro^ (6LU7) of the SARS-CoV2 Coronavirus.

Docking value of Myricetin from *Syzygium aromaticum* exhibits −99.96 kcal/mol and *Gingerenone* from *Zingiber officinale* exhibits −93.9 kcal/mol which comprises of −63.64 and −69.96 van der Waal interactions and −36.32 and −23.94 kcal/mol hydrogen bonding interactions (Figures 4A,B). The residues include hydrogen bonding with the main chain of Arginine at position 188 with binding energy value −5.2 kcal/mol in Myricetin and Leucine at position 287 with binding energy value −5.4 kcal/mol in *Gingerenone* respectively. Park et al. (2013) and Ortega et al. (2017) opined that myricetin exhibits antiviral activity against influenza viruses and anti-HIV-1 activity. It is clear from the above study that myricetin exhibits anti-viral properties against COVID-19. Likewise, Gingerenone A, a polyphenol present in ginger, has attracted increasing attention as potential agents for preventing and treating many oxidative stress-related diseases. No anti-viral report has been published on this compound, but *Zingiber officianle* possessed excellent anti-viral properties (Sharma et al., 2015; Kaushik et al., 2020).

Tinosporinone from *Tinospora cordifolia* exhibits the energy value −83.42 kcal/mol, which contains the −76.55 van der Waal interaction and −6.86 kcal/mol hydrogen bonding interactions (Figure 4C). The inhibitor closely binds with the specific active site making various close contacts with the residues including hydrogen bonding with the main chain of Thyronine at position 199 with binding energy value −7.2 kcal/mol. Sharma et al. (2019) reported that the *Tinospora cordifolia* exhibits potential antiviral properties against chikungunya virus. Synergetic effects of these compounds possess excellent anti-viral properties against viral diseases.

**Figure 5:**
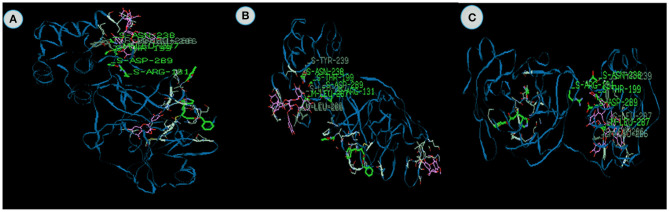
The binding affinities of target compounds **(A)** Nootkatone **(B)** Asarianin and **(C)** gamma- sitosterol present in the Kabasura kudineer (KK) with 3CL^pro^ (6LU7) of the SARS-CoV2 Coronavirus.

Geraniol from *Ceolus ambonicus* (Figure 4D) and Nootkatone (Figure 5A) from *Cypreus rotundus* exhibit the energy value −62.87 and −62.4 kcal/mol which contain the −57.1 and −52.51 van der Waal interactions and −5.6 and −9.8 kcal/mol hydrogen bonding interactions. Lowest binding energy was observed in these two plants. Thyronine 26 and thyronine 111 are the major active sites to bind with 3CL^pro^. Mileva et al. (2015) reported the antiviral properties of Geraniol. Any specific anti-viral report on Nootkatone was not published and it is evident from the investigation that we can use Nootakatone as anti-viral agent for future purposes.

Asarianin (Figure 5B) isolated from *Piper longum* and Gamma sitosterol from *Anacyclus pyrethrum* (Figure 5C) exhibits excellent binding energy against 3CL proviruses and it was found to be −79.94 and −81.94 kcal/mol which comprises of van der Waal interaction and hydrogen bonding interactions. The main active site and domain range of the Asarianin are Thyronine 239 and H-S. Likewise, Alanine 70 and V-M are the active site and binding domain of the 3CL^pro^ on Gammasitosterol. Mair et al. (2016) reported the antiviral activity of piperamides from black pepper. It is the first report that *Anacyclus pyrethrum* possesses antiviral properties on 3CL^pro^ by using *in silico* analysis.

The analysis reports of docking inclusive of the H-bonds that interact with 6LU7 amino acids are tabulated in Table 2. It was observed that every H-bond interacted with the amino acids in the COVID-19 Mpro active site. The presence of H-bonds formed further influences the binding energy. The active site is usually pivotal in analyzing whether the binding site of the ligands has interacted with the amino acids of the target or attached to any other site inside the target. In the present research, the molecular docking analysis affirmed that all the ligands from the natural compounds interacted with the active site of the target protease. The nature and amount of bonding with the active site of the protein decides the higher affinity of drug compounds. Based on the binding energy score, we suggest that Acetoside, Luteolin 7 -rutinoside, rutin, Chebulagic acid, Syrigaresinol, Acanthoside, Violanthin, Andrographidine C, and myricetin exhibit excellent anti-viral properties when compared to synthetic drugs.

Based on the binding energy score, we suggest that these compounds can be tested against Coronavirus and used to develop effective antiviral drugs. Recently, plant compounds such as flavonoids showed good docking affinity against SARS-CoV-2 3CLpro (Jo et al., 2020) and there are several reports on Indian medicinal plants used to treat SARS-CoV infections. In line with this, *Andrographis paniculata* was reported to suppress NLR Family Pyrin Domain Containing 3, caspase-1 and IL-1 β activity on SARS-CoV and likely SARS-CoV-2 virus (Liu et al., 2020a, Liu et al., 2020b). Glycyrrhizin active compound from *Glycyrrhiza glabra* inhibits viral replication of the SARS-associated virus (Cinatl et al., 2003). In addition to their ability to interfere with viral replication, andrographolide from *Andrographis paniculata* exhibits anticancer and immunostimulatory effects (Kumar et al., 2004). Similarly, Jain et al. (2020) reported that the polyherbal formulation of Nilavembu Kudineer has a prophylactic effect and antiviral effect against chikungunya and dengue virus infection. This study hypothesizes that a group of compounds from KK may exert its antiviral properties against novel coronavirus SARS-CoV-2 by either blocking the host cell receptor or inhibiting the key viral protease required for its replication in the host cell. This study might render light on the drug discovery studies for the treatment of viral infections similar to SARS or COVID-19 in future.

## Conclusion

The possible medications using natural compounds were screened from approved bioactive compound databases using molecular docking techniques. This research was aimed at identifying the molecules from natural products that could effectively inhibit the COVID-19 by acting on the main protease (Mpro). Obtained results from molecular docking showed that Acetoside, Luteolin 7 -rutinoside, rutin, Chebulagic acid, Syrigaresinol, Acanthoside, Violanthin, Andrographidine C, myricetin, Gingerenone -A, Tinosporinone, Geraniol, Nootkatone, Asarianin, and sitosterol are main compounds from KK plants which may inhibit COVID-19 giving a better energy score compared to synthetic drugs. Our data suggest these results encourage further *in vitro* and *in vivo* investigations and also encourage traditional use of Kabasura kudineer preventively.

## Data Availability Statement

The original contributions presented in the study are included in the article/Supplementary Materials, further inquiries can be directed to the corresponding author/s.

## Author Contributions

SV and MD conceived and designed the study. SV, MD, SA, and MS carried out the coordination of the study. All authors participated during manuscript development, read, and approved the final version manuscript.

## Conflict of Interest

The authors declare that the research was conducted in the absence of any commercial or financial relationships that could be construed as a potential conflict of interest.
