# Supplementary material for: Molecular Docking Studies on the Anti-viral Effects of Compounds From Kabasura Kudineer on SARS-CoV-2 3CLpro
Source: Front Mol Biosci. 2020 Dec 23;7:613401. doi: 10.3389/fmolb.2020.613401 (PMC7785853; doi:10.3389/fmolb.2020.613401)
Supplement: Supplementary file 1 [file Table_1.docx]

**Molecular docking studies on the anti-viral effects of compounds from Kabasura Kudinneer on SARS- CoV-2 3CL^pro.^**

Savariar Vincent ^a†*^, Selvaraj Arokiaraj^b†^, Muthupandian Saravanan^c†^ and Manoj Dhanraj^a*^

**Supplementary table 1: List of compounds selected from Kabasura Kudineer as ligands**

| **S.No** | **Name of the plant** | **Name of the compound** | **References** |
| --- | --- | --- | --- |
|  | ***Andrographis paniculata*** | Andrographidine C | Bharati *et al*., 2011  Hossain *et al*., 2014 |
|  |  | Andrographidine A |  |
|  |  | Neondrographolide |  |
|  |  | Isoandrographolide |  |
|  |  | andrographolide |  |
|  |  | Bisandrographolide |  |
|  |  | Beta daucosterol |  |
|  |  | Squalene |  |
|  |  | phytol |  |
|  |  | oleonalic acid |  |
|  |  | citronellyl propionate |  |
|  |  | Heptadeconic acid |  |
|  |  | Neophytadine |  |
|  |  | betacarophyllene epoxide |  |
| 2 | ***Adhatoda vasica*** | violanthin | Gangwar and Ghosh, 2014  Singh *et al*., 2011  Kumar *et al*., 2014 |
|  |  | hexadeconic acid |  |
|  |  | sitosterol- |  |
|  |  | epitaxaxerol |  |
|  |  | vasicol |  |
|  |  | vasicinol |  |
|  |  | vasicinolone |  |
|  |  | vasicinone |  |
|  |  | vasicine |  |
|  |  | deoxyvasicinone |  |
|  |  | pentadecene |  |
|  | ***Clerodendrum serratum*** | Acetoside | Patel *et al*., 2014  Kumar and Nishteswar, 2013  Noreen *et al*., 2018  Maajan *et al*., 2019 |
|  |  | Monomelittoside |  |
|  |  | 6-Hydroxyluteolin 7-glucoside |  |
|  |  | Hispidulin |  |
|  |  | scutellarein |  |
|  |  | pectoliniarigenin |  |
|  |  | Beta sitosterol |  |
|  |  | Queretaroic acid |  |
|  |  | Serratagenic acid |  |
|  |  | alpha-Spinasterol |  |
|  |  | Friedelin |  |
|  |  | Oleanolic acid |  |
|  | ***Ceolus ambonicus*** | Geraniol | Pillai *et al*., 2011  Arumugam *et al*., 2016  Rout *et al*., 2012  Bhattacharjee, 2010 |
|  |  | Thymol |  |
|  |  | Cavracol |  |
|  |  | Spathulenol |  |
|  |  | Alpha murrolene |  |
|  |  | Alpha humelene |  |
|  |  | Beta patucholene |  |
|  |  | Beta carophyllene |  |
|  |  | Beta selinene |  |
|  |  | Gamma terpinine |  |
|  | ***Cypreus rotundus*** | Nootkatone | Peerzada *et al*., 2015  Al- Snafi, 2016  Huang,1999 |
|  |  | Beta selinene |  |
|  |  | Valencene |  |
|  |  | Crophyllene oxide |  |
|  |  | Trans pinocarveol |  |
|  |  | Alpha longipinane |  |
|  |  | Patchoulrnone |  |
|  |  | Cuprene |  |
|  | ***Hygrophila auricualata*** | Luteolin -7 rutinoside | Hussain*et al*., 2010  Sarvananda and Premarathna, 2018  Salve and Bhuktar, 2017 |
|  |  | Apigenin 7- glucronide |  |
|  |  | Luteolin |  |
|  |  | Betulin |  |
|  |  | Histidine |  |
|  |  | Glucornic acid |  |
|  |  | Syringic acid |  |
|  |  | Phenylalanine |  |
|  |  | L- Rhamnose |  |
|  |  | Lupenone |  |
|  | ***Sausurea lappa*** | Syringaresinol | Singh *et al*., 2017  Madhuri *et al*., 2012  Liu *et al*., 2012  Robinson *et al*., 2008 |
|  |  | Lappidilactone |  |
|  |  | Saussureamine |  |
|  |  | Cynaropicrin |  |
|  |  | scopoletin |  |
|  |  | Betulinic acid ethyl ester |  |
|  |  | costunolide |  |
|  |  | dehydrocostus lactone |  |
|  |  | Soulangianolide A |  |
|  | ***Syzygium aromaticum*** | Myricetin | Jimoh *et al*., 2017  Kaur and Kaushal, 2019  Batiha *et al*., 2020 |
|  |  | Biflorin |  |
|  |  | Kaempeferol |  |
|  |  | Stigmasterol |  |
|  |  | Eugenol acetate |  |
|  |  | Eugenol |  |
|  |  | Alpha copaene |  |
|  |  | Gamma murolene |  |
|  |  | Beta selinene |  |
|  |  | Carophyllene oxide |  |
|  |  | Valencene |  |
|  |  | Alpha humelene |  |
|  |  | Germacrene |  |
|  |  | Beta carophyllene |  |
|  |  | Iso carophyllene |  |
|  | ***Sida acuta*** | Syrigaresinol | Karou *et al*., 2007  Obah *et al*., 2007  Tcheghebe *et al*., 2017  Jindal *et al*., 2012 |
|  |  | Acanthoside |  |
|  |  | Alpha tocopherol |  |
|  |  | Evofolin |  |
|  |  | 4-Ketopinoresinol |  |
|  |  | glucopyranoside |  |
|  |  | scopoletin |  |
|  |  | Campesterol |  |
|  |  | Taraxasterone |  |
|  |  | cryptolepine |  |
|  |  | Vomifoliol |  |
|  |  | 11-Methoxy-10H-quindoline |  |
|  |  | alpha amyrin |  |
|  |  | Loliolide |  |
|  | ***Tinospora cordifolia*** | Tinosporinone | Upadhyay *et al*., 2010  Rout, 2006  Patel *et al*., 2009  Gupta *et al*., 2011  Jgetia *et al*., 2006  Patel *et al*., 2011  Tiwari *et al*., 2018 |
|  |  | Palmatine |  |
|  |  | Berberine |  |
|  |  | Tinosporide |  |
|  |  | Jatorhizine |  |
|  |  | Columbin |  |
|  |  | Tinocordiofolin |  |
|  | ***Terminalia chebula*** | Chebulagic acid | Rathinamoorthy and Thilagavathi, 2014  Ashwini *et al*., 2011  Upadhyay *et al*., 2014 |
|  |  | Chebulinin Acid |  |
|  |  | Rutin |  |
|  |  | Punicalin |  |
|  |  | Quercetin |  |
|  |  | Pelargonodin |  |
|  |  | Ellagic acid |  |
|  |  | Arjungenin |  |
|  |  | Rjunic acid |  |
|  |  | arjunetin |  |
|  | ***Tragia involucrata*** | Rutin | Gobalakrishnan *et al*., 2013  Mothana *et al*., 2010  Dhara *et al*., 2000  Rao *et al*., 2007 |
|  |  | Quercetin |  |
|  |  | Stigmasterol |  |
|  |  | Shellsol |  |
|  |  | 5-Hydroxy-1-methylpiperidin-2-one |  |
|  | ***Zingiber officianle*** | Gingerenone - A | Rahmani, 2014  Amir *et al*., 2011  Marrelli *et al*., 2015 |
|  |  | Gingerol |  |
|  |  | 6- dehydrogingerdione |  |
|  |  | Paradol |  |
|  |  | Shagol |  |
|  |  | beta sesquiphellendrane |  |
|  |  | Zingerone- |  |
|  |  | alpha farnesene |  |
|  |  | alpha curcumene |  |
|  |  | Bisabolene |  |
|  |  | Zerumbone |  |
|  | ***Piper longum*** | Asarinin | Zaveri *et al*., 2010  Kumar *et al*., 2011  Khushbu *et al*., 2011 |
|  |  | Sesamin |  |
|  |  | Coumaperine |  |
|  |  | Piperolctam |  |
|  |  | Piperine |  |
|  | ***Anacyclus pyrethrum*** | Gamma sitosterol | Selles *et al*., 2012  Selles *et al*., 2013  Usmnai *et al*., 2016 |
|  |  | Triisobutyl(3-phenylpropoxy)silane |  |
|  |  | 9,12-Octadecadienoic acid (Z,Z) |  |
|  |  | Palmitiacid |  |
|  | **Control drug** | Azithromycin  Oseltamivir  Ritonavir  HCQ  Ivermectin  Lopinavir, | Gautret *et al*., 2020  Muralidharan *et al*., 2020  Cao *et al*., 2020  Velavan and Meyer, 2020  Caly *et al*., 2020 |

**Supplementary table 2: Molecular Docking studies on the few Phytocompounds from Kabasura kudineer on 3CL^pro^  (6LU7) (SARS-CoV-2 ) using iGEMDOCK software**

| **S.NO** | **Name of the plant** | **Name of the compound** | **Energy value** | **Binding Domain and Aminoacids** |
| --- | --- | --- | --- | --- |
| 1 | ***Andrographis paniculata*** | **Andrographidine C** | **-98.5** | H-M-ALA-2  V-M-LEU-167  V-M-PRO-168 V-S-PRO-168  V-M-THR-190 V-M-ALA-2 |
|  |  | Andrographidine A | -90.3 |  |
|  |  | Neondrographolide | -90.1 |  |
|  |  | Isoandrographolide | -86.5 |  |
|  |  | andrographolide | -83.9 |  |
|  |  | Bisandrographolide | -83.7 |  |
|  |  | Beta daucosterol | -74.2 |  |
|  |  | Squalene | -71 |  |
|  |  | phytol | -64.6 |  |
|  |  | oleonalic acid | -60 |  |
|  |  | citronellyl propionate | -59.7 |  |
|  |  | Heptadeconic acid | -58.5 |  |
|  |  | Neophytadine | -53.5 |  |
|  |  | betacarophyllene epoxide | -42.5 |  |
| 2 | ***Adhatoda vasica*** | **violanthin** | **-96.8** | H-S-GLU-166  H-M-LEU-167 V-M-LEU-167 V-M-PRO-16 V-S-PRO-168 |
|  |  | hexadeconic acid | -70.8 |  |
|  |  | sitosterol- | -69.2 |  |
|  |  | epitaxaxerol | -67.2 |  |
|  |  | vasicol | -63.8 |  |
|  |  | vasicinol | -61.8 |  |
|  |  | vasicinolone | -59.7 |  |
|  |  | vasicinone | -56.7 |  |
|  |  | vasicine | -56.1 |  |
|  |  | deoxyvasicinone | -53.2 |  |
|  |  | pentadecene | -52.1 |  |
| 3 | ***Clerodendrum serratum*** | **Acetoside** | **-111.4** | V-M-PRO-168 V-S-PRO-168  V-M-THR-190 V-M-ALA-2 |
|  |  | Monomelittoside | -101.8 |  |
|  |  | 6-Hydroxyluteolin 7-glucoside | -95.5 |  |
|  |  | Hispidulin | -83.5 |  |
|  |  | scutellarein | -83.5 |  |
|  |  | pectoliniarigenin | -78.7 |  |
|  |  | Beta sitosterol | -77.3 |  |
|  |  | Queretaroic acid | -69.9 |  |
|  |  | Serratagenic acid | -67.3 |  |
|  |  | alpha-Spinasterol | -61.9 |  |
|  |  | Friedelin | -54.2 |  |
|  |  | Oleanolic acid | -50.8 |  |
| 4 | ***Ceolus ambonicus*** | Geraniol | -55.6 | V-M-LEU-167 V-M-PRO-168 V-S-PRO-168  V-M-ALA-2 |
|  |  | Thymol | -49.1 |  |
|  |  | Cavracol | -48.4 |  |
|  |  | Spathulenol | -46.6 |  |
|  |  | Alpha murrolene | -45.8 |  |
|  |  | Alpha humelene | -43 |  |
|  |  | Beta patucholene | -42.6 |  |
|  |  | Beta carophyllene | -41.9 |  |
|  |  | Beta selinene | -41.9 |  |
|  |  | Gamma terpinine | -37.6 |  |
| 5 | ***Cypreus rotundus*** | Nootkatone | -49.9 | V-M-PRO-168 V-S-PRO-168 |
|  |  | Beta selinene | -43.7 |  |
|  |  | Valencene | -42.7 |  |
|  |  | Crophyllene oxide | -42.2 |  |
|  |  | Trans pinocarveol | -39.7 |  |
|  |  | Alpha longipinane | -38.9 |  |
|  |  | Patchoulrnone | -36 |  |
|  |  | Cuprene | -34.7 |  |
| 6 | ***Hygrophila auricualata*** | Luteolin -7 rutinoside | -99.9 | H-M-ALA-2  V-M-PRO-168 V-S-PRO-168 |
|  |  | Apigenin 7- glucronide | -89.1 |  |
|  |  | Luteolin | -82.1 |  |
|  |  | Betulin | -66.8 |  |
|  |  | Histidine | -62.6 |  |
|  |  | Glucornic acid | -59.1 |  |
|  |  | Syringic acid | -59.1 |  |
|  |  | Phenylalanine | -54.4 |  |
|  |  | L- Rhamnose | -52.8 |  |
|  |  | Lupenone | -51.4 |  |
| 7 | ***Sausurea lappa*** | Syringaresinol | -81.2 | H-M-ALA-2  V-M-PRO-168 V-S-PRO-168  V-M-THR-190 V-M-ALA-2 |
|  |  | Lappidilactone | -76.3 |  |
|  |  | Saussureamine | -76.1 |  |
|  |  | Cynaropicrin | 75.7 |  |
|  |  | scopoletin | -63.3 |  |
|  |  | Betulinic acid ethyl ester | -59.4 |  |
|  |  | costunolide | -4.2 |  |
|  |  | dehydrocostus lactone | -43.1 |  |
|  |  | Soulangianolide A | -39.9 |  |
| 8 | ***Syzygium aromaticum*** | Myricetin | -85 | H-M-ALA-2 V-M-LEU-167 V-M-PRO-168 V-S-PRO-168 V-M-ALA-2 |
|  |  | Biflorin | -83.4 |  |
|  |  | Kaempeferol | -77.8 |  |
|  |  | Stigmasterol | -68.3 |  |
|  |  | Eugenol acetate | -54.5 |  |
|  |  | Eugenol | -51.5 |  |
|  |  | Alpha copaene | -45.9 |  |
|  |  | Gamma murolene | -44.6 |  |
|  |  | Beta selinene | -43.7 |  |
|  |  | Carophyllene oxide | -42.7 |  |
|  |  | Valencene | -42.7 |  |
|  |  | Alpha humelene | -42.4 |  |
|  |  | Germacrene | -42 |  |
|  |  | Beta carophyllene | -39.6 |  |
|  |  | Iso carophyllene | -39.6 |  |
| 9 | ***Sida acuta*** | Syrigaresinol | -100.9 | H-M-ALA-2  V-M-PRO-168 V-S-PRO-168  V-M-THR-190 V-M-ALA-191 V-M-ALA-2 |
|  |  | Acanthoside | -98.1 |  |
|  |  | Alpha tocopherol | -82.8 |  |
|  |  | Evofolin | -77.7 |  |
|  |  | 4-Ketopinoresinol | -73.4 |  |
|  |  | glucopyranoside | -66.7 |  |
|  |  | scopoletin | -63.3 |  |
|  |  | Campesterol | -60.9 |  |
|  |  | Taraxasterone | -58.5 |  |
|  |  | cryptolepine | -56.8 |  |
|  |  | Vomifoliol | -55.6 |  |
|  |  | 11-Methoxy-10H-quindoline | -53.4 |  |
|  |  | alpha amyrin | -5.3 |  |
|  |  | Loliolide | -47.3 |  |
| 10 | ***Tinospora cordifolia*** | Tinosporinone | -81.5 | H-M-ALA-2  V-M-PRO-168 V-S-PRO-168 V-M-ALA-2 |
|  |  | Palmatine | -68.8 |  |
|  |  | Berberine | -66.8 |  |
|  |  | Tinosporide | -63.6 |  |
|  |  | Jatorhizine | -63.3 |  |
|  |  | Columbin | -61.7 |  |
|  |  | Tinocordiofolin | -48.5 |  |
| 11 | ***Terminalia chebula*** | Chebulagic acid | -114.4 | H-M-ALA-2  V-M-PRO-168 V-S-PRO-168  V-M-THR-190 |
|  |  | Chebulinin Acid | -110.1 |  |
|  |  | Rutin | -97.9 |  |
|  |  | Punicalin | -85.8 |  |
|  |  | Quercetin | -82.3 |  |
|  |  | Pelargonodin | -77 |  |
|  |  | Ellagic acid | 68.5 |  |
|  |  | Arjungenin | -56.1 |  |
|  |  | Rjunic acid | -56.1 |  |
|  |  | arjunetin | -52.9 |  |
| 12 | ***Tragia involerta*** | Rutin | -99.5 | V-M-PRO-168 V-S-PRO-168 |
|  |  | Quercetin | -81.1 |  |
|  |  | Stigmasterol | -61.3 |  |
|  |  | Shellsol | -43.1 |  |
|  |  | 5-Hydroxy-1-methylpiperidin-2-one | -41 |  |
| 13 | ***Zingiber officianle*** | Gingerenone - A | -74.5 | H-M-ALA-2  V-M-PRO-168 V-S-PRO-168  V-M-ALA-2 |
|  |  | Gingerol | -73.8 |  |
|  |  | 6- dehydrogingerdione | -67.1 |  |
|  |  | Paradol | -66.9 |  |
|  |  | Shagol | -66.1 |  |
|  |  | beta sesquiphellendrane | -56.3 |  |
|  |  | Zingerone- | -55 |  |
|  |  | alpha farnesene | -54.8 |  |
|  |  | alpha curcumene | -52.2 |  |
|  |  | Bisabolene | -48.8 |  |
|  |  | Zerumbone | -34.3 |  |
| 14 | ***Piper longum*** | Asarinin | -70.2 | H-M-ALA-2  V-S-PRO-168 |
|  |  | Sesamin | -70 |  |
|  |  | Coumaperine | -64.4 |  |
|  |  | Piperolctam | -59.7 |  |
|  |  | Piperine | -59.2 |  |
| 15 | ***Anacyclus pyrethrum*** | Gamma sitosterol | -67.6 | V-S-PRO-168 |
|  |  | Triisobutyl(3-phenylpropoxy) silane | -67.2 |  |
|  |  | 9,12-Octadecadienoic acid (Z, Z) | -61.3 |  |
|  |  | palmitiacid | -58.6 |  |
| 16. | **Control drug** | Ritonavir | -87.2 | H-M-GLU-166  H-M-GLN-189  H-S-ARG-188 |
|  |  | Lopinavir | -85.1 |  |
|  |  | Oseltamivir | -75 |  |
|  |  | HCQ | -70.1 |  |
|  |  | Ivermectin | -54.6 |  |
|  |  | Azithromycin - | -43.2 |  |

*** The possible binding modes of selected phytochemicals at the target protein active sites.**

**H-S signifies hydrogen bond with sidechain;**

**H-M signifies hydrogen bond with the main chain**

**V-M signifies Vander waals interaction with main chain**

**V-S signifies Vander waals interaction with side chain**
