# Supplementary material for: Molecular Docking Studies on the Anti-viral Effects of Compounds From Kabasura Kudineer on SARS-CoV-2 3CLpro
Source: Front Mol Biosci. 2020 Dec 23;7:613401. doi: 10.3389/fmolb.2020.613401 (PMC7785853; doi:10.3389/fmolb.2020.613401)
Supplement: Supplementary file 2 [file Image_1.pdf]

Molecular docking studies on the anti-viral effects of compounds from Kabasura Kudinneer on SARS- CoV-2 3CL$^{pro.}$

Savariar Vincent [a†*], Selvaraj Arokiaraj[b†], Muthupandian Saravanan[c†] and Manoj Dhanraj[a*]

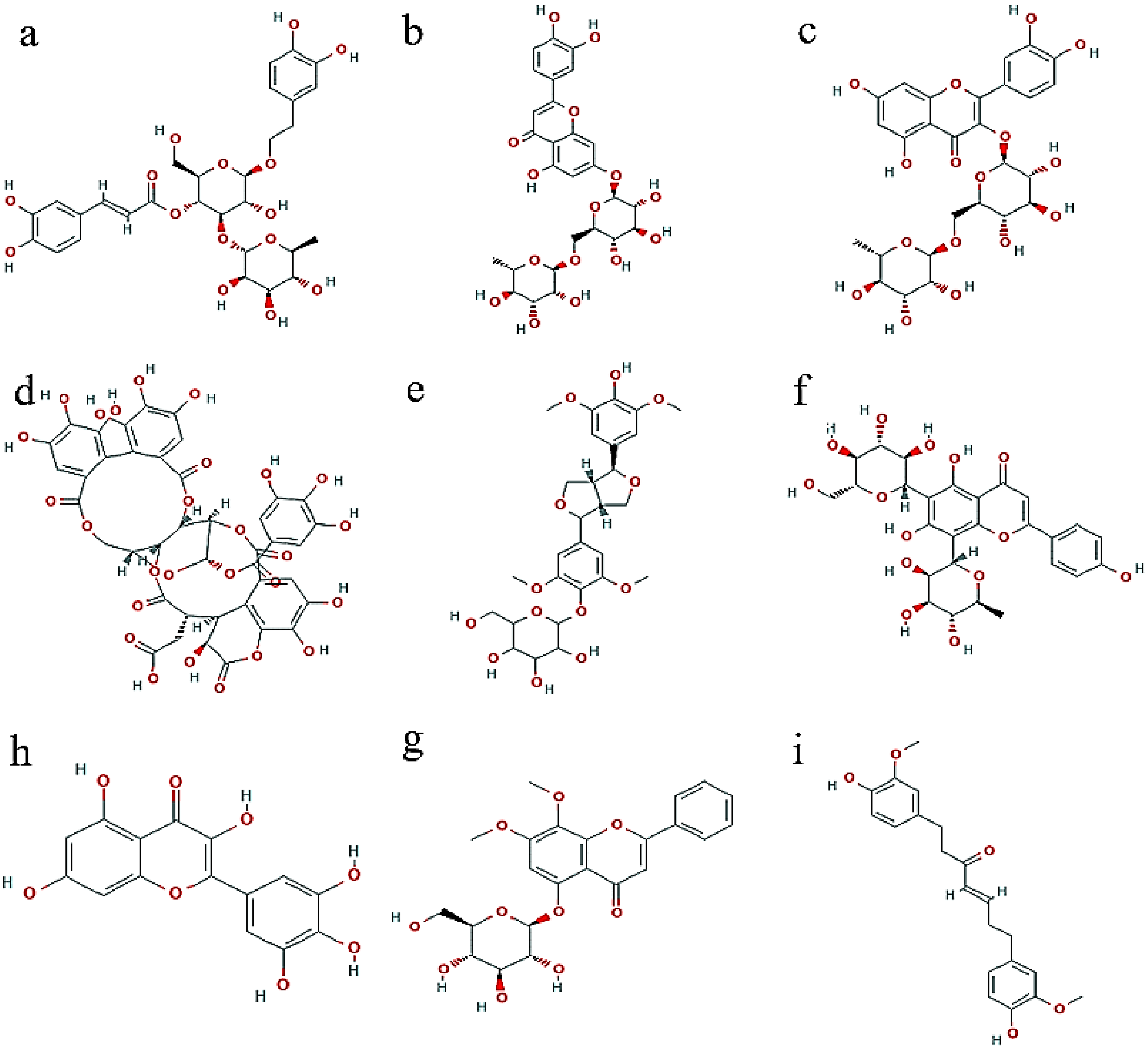

Supplementary Fig. 1 Structures of target compounds in the Kabasura Kudineer medicinal plants. Source: https://pubchem.ncbi.nlm.nih.gov/ (a) Acetoside, (b) Luteolin 7 –rutinoside, (c) Rutin (d) Chebulagic acid, (e) Acanthoside, (f) Syrigaresinol, (g) Violanthin, (h) Andrographidine C, (i) Myricetin

.

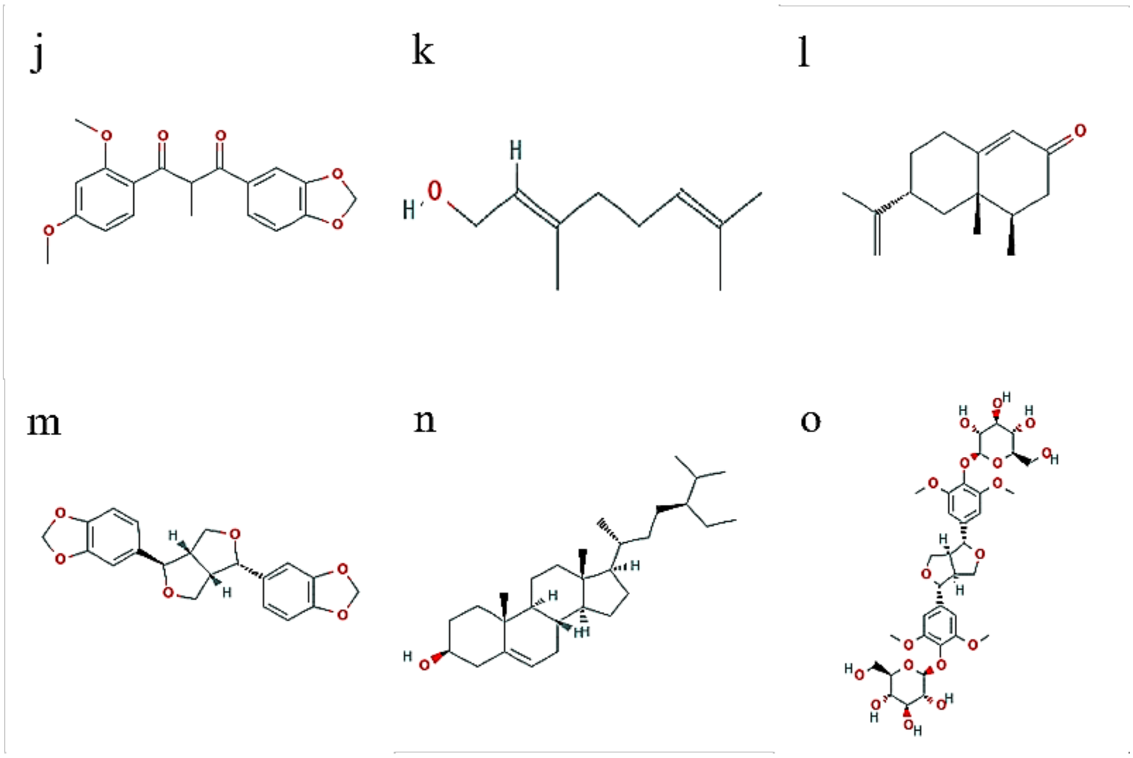

Supplementary Fig. 1 Structures of Target compounds in the Kabasura Kudineer medicinal plants. Source: https://pubchem.ncbi.nlm.nih.gov/ (j) Gingerenone -A, (k) Tinosporinone, (l) Geraniol, (m) Nootkatone, (n) Asarianin, (o) Gamma sitosterol
